# Observational Application Comparing Problem-Based Learning with the Conventional Teaching Method for Clinical Acupuncture Education

**DOI:** 10.1155/2019/2102304

**Published:** 2019-03-18

**Authors:** Yun Jin Kim

**Affiliations:** School of Traditional Chinese Medicine, Xiamen University Malaysia, Jalan Sunsuria, Bandar Sunsuria, Sepang, Selangor 43900, Malaysia

## Abstract

**Aim:**

Although the problem-based learning (PBL) teaching method was introduced in 1969, its rapid and widespread application in Malaysia started in 1979. This study aimed to evaluate satisfaction with PBL compared to that of conventional learning, using satisfaction surveys and the Rosenberg Self-Esteem scores, of students learning clinical acupuncture at the School of Traditional Chinese Medicine (TCM), Xiamen University Malaysia.

**Method:**

The participants of this study (N=36) were registered for a bachelor's degree program in TCM in 2016 and enrolled in the Science of Acupuncture and Moxibustion course beginning in September 2018. The students were randomly allocated into two groups: PBL group and conventional group. A self-administered learning satisfaction survey and the Rosenberg Self-Esteem scores were used for data collection. An independent sample* t*-test was used to compare the results between the two groups. A* p*-value <0.05 was considered significant.

**Results:**

The results of the learning satisfaction survey and Rosenberg Self-Esteem scores were significantly better in the PBL group than in the conventional group (*p*<0.05).

**Conclusions:**

PBL appears to be more effective for clinical acupuncture education than the conventional teaching method. However, further studies are needed to identify the mechanisms by which PBL excels in clinical acupuncture education, as well as other related TCM fields.

## 1. Introduction

Barrows and Tamblyn first introduced the problem-based learning (PBL) teaching method at McMaster University in 1969. This method has since become a popular model for medical education worldwide. There are five key categories of PBL, namely, cooperative learning, self-determination, information processing, problem-solving, and contextual learning [1].

Recently, modern medical educational systems have attempted to incorporate teaching methods beneficial for medical students' learning, in regard to their critical thinking, clinical practice skills training, medical knowledge achievement, and continued medical professional learning [2]. The use of PBL, along with educational workplace collaboration and interdisciplinary medical learning, has spread beyond the traditional realm of clinical medicine education into related areas, such as health sciences and biomedical engineering. With the growth of such medical practices, the popularity of PBL in various medical educational and organizational settings has increased, and a number of studies have assessed its effectiveness on the quality of medical students' learning and the development and improvement of self-learning situations [3].

In Malaysia, the use of PBL in medical schools started at the Universiti Sains Malaysia (USM) in 1979. PBL is currently used by all medical schools in Malaysia, because it is a requirement for educational accreditation by the Malaysia Qualifications Agency (MQA) and the Malaysian Medical Council (MMC) [4]. Malaysia's Ministry of Higher Education (MOHE) has suggested that public and private universities utilize PBL in their teaching strategies and plans, which is a promising creative innovation in Malaysian higher education settings [5].

Another field of medicine that has adopted PBL is clinical acupuncture education. Acupuncture is a simple and effective procedure, used worldwide, that has less adverse effects than other forms of treatment, such as herbal medicine [6]. PBL is an appropriate tool for teaching the subspecializations of clinical acupuncture, since most of the PBL scenarios are based on the systematic teaching method. Also, in contrast to conventional clinical acupuncture education and clinical skills training methods, PBL is a student-centred teaching approach, in which students develop clinical reasoning skills, identify their learning needs in an interactive group discussion, apply newly gained knowledge to problems, and summarize what they learn with the group. There are many advantages to the integration of PBL using clinical case scenarios in acupuncture education and clinical skills training. In this way, students learn the topic by experiencing a clinical case and discussing it in a tutor-guided small group. This method also helps students with the use of self-evaluations. Additionally, PBL allows students to acquire basic knowledge of acupuncture and the Meridian Theory and to learn clinical skills. PBL is helpful for cultivating clinical thinking skills and enhancing continued medical professional learning programmes.

Therefore, the aim of this study was to evaluate learning satisfaction from the students' points of view, using a satisfaction survey and the Rosenberg Self-Esteem Score, of PBL compared with that of conventional learning for clinical acupuncture.

## 2. Materials and Methods

### 2.1. Study Design

The participants of this study (N=36) were enrolled in a bachelor's degree program in Traditional Chinese Medicine (TCM) in 2016, and the study was conducted in the Science of Acupuncture and Moxibustion course (Course Code: TCM303) beginning in September 2018 at the School of TCM, Xiamen University Malaysia.

According to the local and national ethical instructions for research (National Committee for Clinical Research: http://www.nccr.gov.my/index.cfm) guidelines, this study did not require ethical approval. The human biological tissues, good clinical practice (GCP), and clinical trial research subjects were respected. All students were adequately informed about the purpose and granted anonymity and confidentiality regarding their data. We obtained written informed consent from all students prior to study participation.

We randomly allocated the participants into two groups, the PBL group and the conventional group, each containing 18 students. The PBL group was further subdivided into three groups, with six students in each group. The PBL course included Phase A modules that were designed to measure the student's clinical knowledge of acupuncture treatment strategies, using case reports, and Phase B modules that were designed to discuss decision-making regarding medical ethics and cultural dimensions of traditional and complementary medical care in Malaysia, China, and other countries. Both phases aimed to teach students the ability to integrate their knowledge of acupuncture in learning clinical practice skills and discussing medical ethics. Each group of six students worked together to collect information, discuss possible mechanisms and causes, develop hypotheses and strategies to test the hypotheses, and prepare for presentations and discussions in class.

The conventional group was taught with teacher-centred learning, involving lecturers and short discussions. While the problem-solving element was presented by and/or discussed with the lecturer in class, the teaching plan and materials were determined by the lecturer and conveyed to the students during lecture.

### 2.2. Study Procedure

The PBL and conventional teaching plans were created by the School of TCM, Xiamen University Malaysia's expert panel, consisting of an external examiner and an industrial advisor. Each teaching plan included learning outcomes, clinical case reports exhibiting medical ethical dilemmas, and guidelines for lecturers, and expert external panels. The assessments used by both groups consisted of the following: (a) the learning satisfaction survey and (b) the Rosenberg Self-Esteem Scale. At the end of six sessions, the lecturer was instructed to leave the lecture hall, and all students completed the learning satisfaction survey and Rosenberg Self-Esteem Scale. The forms were then collected in a ballot box.

The research process consisted of three steps as follows:

*Step 1*

1. Setting up the curriculum for peer lecturers for the PBL and conventional teaching methods. The teaching components of the curriculum included acupuncture basic theories and clinical case studies, the acupuncturist-patient relationship, and rights and responsibilities of patients and acupuncturists. Each teaching plan was designed with learning objectives, learning outcomes, clinical case studies, and course guidelines for the lecturer. For the PBL method, the course guidelines included details of the PBL steps and potential ethical problems for the clinical case studies.
2. Setting up the learning satisfaction questions. External panels for content validity provided 10 questions, which were measured on a 5-point scale. Learning satisfaction was measured by the students' subjective feelings. Students' satisfaction/feelings were coded on a 4-point scale, ranging from 1 (strongly disagree) to 4 (strongly agree).
3. Adopting the Rosenberg Self-Esteem Scale. The Rosenberg Self-Esteem Scale assesses a student's overall evaluation of personal worthiness as a human being. The scale consisted of 10 Likert-type scale items designed to assess positive and negative evaluations of self. Students' responses were coded on a 4-point scale, ranging from 1 (strongly disagree) to 4 (strongly agree). Thus, a possible total score ranged from 10 to 40, with higher scores reflecting more positive evaluations of self-esteem [7].

*Step 2*

1. Pretest using the Rosenberg Self-Esteem Scale. All students were tested and scored on this scale before classes began.
2. Conducting the education. Each group was assigned a separate classroom. The courses were taught or supervised by the same lecturer for six sessions. The teaching in the conventional group was teacher-centred, although there was some discussion after the lecture. Both groups received an introduction to the course in the first session.

*Step 3*

1. Posttest using the Rosenberg Self-Esteem Scale. All students were tested and scored on this scale after the course ended.
2. Learning satisfaction survey. All students answered the learning satisfaction questions after the course ended, and the answers were scored.

### 2.3. Statistical Analysis

Data collection and statistical analysis were performed using SPSS software (version 16.0; IBM, USA) for data analysis. All data are shown as mean ± standard deviation (SD). An independent sample* t*-test was used to compare results between the PBL and the conventional groups. A* p*-value of <0.05 was considered statistically significant.

## 3. Results

**Figure 1:**
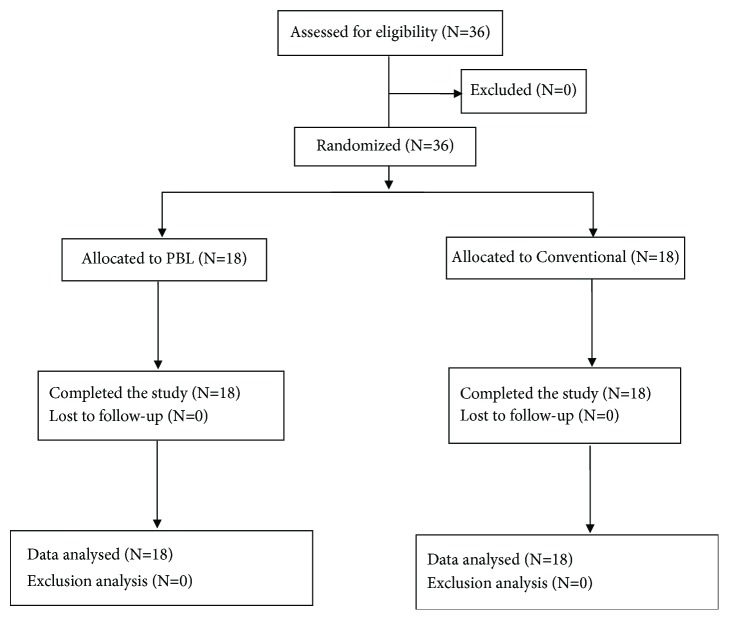
The flow of the research design.

Thirty-six participants were present at the initial session, and all 36 entered the study and were equally allocated into the PBL and conventional groups, including 18 participants in each group. All 36 participants completed the satisfaction survey and Rosenberg Self-Esteem Scales. The study flow diagram is shown in Figure 1.

There were no statistically significant differences in sociodemographic variables between the two groups (Table 1).

On the learning satisfaction survey completed at the end of the six sessions, the PBL group demonstrated significantly higher satisfaction levels regarding clinical acupuncture knowledge (*p*=0.021), improvement of medical interviewing skills (*p*=0.035), interest in the topic and enjoyment of learning (*p*=0.017), self-directed learning of the subject material (*p*=0.019), collaboration in small groups (*p*=0.015), learning outcome (*p*=0.018), and understanding of patient evaluation and management (*p*=0.039). However, the two groups were not statistically different regarding basic acupuncture knowledge, including preclinical to clinical knowledge and and improvement of hands-on skills (Table 2).

On the Rosenberg Self-Esteem Scale, comparing the pre- and posteducation scores, the PBL group demonstrated significant improvement on item 1 (*p*=0.021), item 3 (*p*=0.012), item 4 (*p*=0.038), item 5 (*p*=0.036), item 7 (*p*=0.024), item 8 (*p*=0.015), item 9 (*p*=0.025), and item 10 (*p*=0.019). The conventional group demonstrated significant improvement on item 1 (*p*=0.025), item 3 (*p*=0.017), and item 7 (*p*=0.023), as shown in Table 3.

## 4. Discussion

Currently, the implementation of PBL in Malaysian medical schools has been extensive. The major objectives of PBL evaluated in this study were the acquisition of relevant medical knowledge, clinical skills, medical interviewing skills, and medical ethics in a clinical acupuncture context, which allows students to obtain information from a wide range of viewpoints [8]. PBL motivates students to think critically and generate ideas and to acquire the basic medical and clinical knowledge, clinical skills, and behaviour required to become competent clinical physicians [9]. Another study showed that for the PBL sessions were observed regarding the availability of enough learning resource [10].

PBL uses a “focus on the problem” approach. The starting point is realistic and helps students learn to contextualize problems within a scenario. PBL is a student-centred, small group-based, learning approach and presents challenging problems based upon realistic situations. Based on constructivist epistemology, PBL begins with an unstructured problem that has multiple possible solutions [11]. The students participate in self-directed learning and apply their new knowledge to the problem. They then reflect on their learning and the effectiveness of their chosen solution. PBL is a useful educational methodology for TCM students in that students are expected to understand basic medical concepts in the context of clinical cases, which supports the recollection and application of their knowledge and case-based problem-solving skills later in their professional careers [12].

The Rosenberg Self-Esteem Scale is a well-known questionnaire, introduced in 1965 by Rosenberg and is the most widely used method for the measurement of self-esteem, which is a person's overall evaluation of worthiness as a human being [13]. Self-esteem is a central construct in clinical, personality, and social psychology. Its role in human psychological functioning has been studied for more than a century [14]. Self-esteem is related to personal beliefs about skills, abilities, and social relationships [15]. People with higher self-esteem are more likable and attractive, they have better relationships, and they make better impressions on others than those with lower self-esteem. Encouraging a person's self-esteem provides positive effects on the person's communication skills and ongoing self-directed learning outcome.

Our study compared the PBL method with conventional learning using a satisfaction survey and the Rosenberg Self-Esteem Scale. When the PBL method was used, students demonstrated significantly higher levels of satisfaction with learning outcomes, as well as higher self-esteem scores, than students taught with the conventional method [16].

## 5. Conclusions

This study's results demonstrate that PBL is preferable for clinical acupuncture education over the conventional method. PBL is an effective approach, especially for the retention and application of clinical acupuncture knowledge.

However, further studies are needed to uncover the mechanisms by which PBL is effective in clinical acupuncture education. Such information might improve the PBL skills of the lecturer, thus making the curriculum more flexible and allowing for the integration of basic acupuncture theory to clinical acupuncture education. With a broader application of PBL, more students could focus on solving clinical problems in clinical-related courses.

## Figures and Tables

**Table 1:** Sociodemographic comparison between participants in the PBL and conventional groups.

Index		PBL	Conventional	*p-*value
Gender n (%)	Male	10 (55.5%)	9 (50%)	0.328
	Female	8 (44.5%)	9 (50%)	

Nationality n (%)	Malaysia	12 (66.7%)	13 (72.2%)	0.167
	China	6 (33.3%)	5 (27.8%)	
	Others	0 (0%)	0 (0%)	

Previous degree n (%)	STPM	6 (33.3%)	9 (50%)	0.381
	UEC	9 (50%)	7 (39%)	
	A Level	1 (5.7%)	0 (0%)	
	Foundation	2 (11%)	2 (11%)	

Scholarship n (%)	Y	6 (33.3%)	7 (39%)	0.513
	N	12 (66.7%)	11 (61%)	

Financial stress n (%)	Y	2 (11%)	1 (5.7%)	0.512
	N	16 (89%)	17 (94.3%)	

Physical problem n (%)	Y	2 (11%)	1 (5.7%)	0.316
	N	16 (89%)	17 (94.3%)	

Mental health n (%)	Y	0 (0%)	0 (0%)	0.258
	N	18 (100%)	18 (100%)	

First language n (%)	Chinese	17 (94.3%)	16 (89%)	0.552
	English	1 (5.7%)	2 (11%)	
	Bahasa Malay	0 (0%)	0 (0%)	

**Table 2:** Results from the learning satisfaction surveys of students in the PBL and conventional groups.

Questions	PBL	Conventional	*p*-value
(1) Satisfaction of basic acupuncture knowledge	3.44±0.83	3.38±0.13	0.216
(2) Satisfaction of clinical acupuncture knowledge	3.23±0.57^*∗*^	2.13±0.08	0.021
(3) Satisfaction of pre-clinical to clinical knowledge	3.89±0.35	3.78±0.12	0.191
(4) Satisfaction of improvement of hands-on skills	3.57±0.34	3.14±0.32	0.258
(5) Satisfaction of improvement of medical interviewing skills	3.98±0.13^*∗*^	1.76±0.11	0.035
(6) Satisfaction of interest in the topic and enjoyment of learning	3.46±0.59^*∗*^	1.03±0.45	0.017
(7) Satisfaction of self-directed learning of the subject matter	3.22±0.75^*∗*^	1.32±0.50	0.019
(8) Satisfaction of collaboration in small groups	3.48±0.27^*∗*^	1.64±0.36	0.015
(9) Satisfaction of learning outcome	3.92±0.15^*∗*^	2.78±0.59	0.018
(10) Satisfaction of understanding of patient evaluation and management	3.23±0.21^*∗*^	1.89±0.64	0.039

Values represent mean ± SD.

A higher score indicates a better outcome.

^*∗*^Indicates a statistical difference from the conventional group (*p*<0.05).

**Table 3:** Results of the pre- and posteducation Rosenberg Self-Esteem Scale in the PBL and conventional groups.

Items	PBL			Conventional		
	Pre-	Post-	*p-* value	Pre-	Post-	*p- *value
(1) On the whole, I am satisfied with myself	1.12±0.12	2.97±0.13^*∗*†^	0.021	1.13±0.11	2.02±0.13^†^	0.025
(2) At times, I think I am no good at all	3.54±0.23	2.78±0.17	0.066	2.87±0.15	2.77±0.17	0.083
(3) I feel that I have a number of good qualities	1.31±0.33	2.78±0.18^*∗*†^	0.012	1.34±0.09	1.88±0.32^†^	0.017
(4) I am able to do things as well as most other people	1.43±0.22	2.68±0.14^*∗*†^	0.038	1.38±0.11	1.78±0.22	0.071
(5) I feel I do not have much to be proud of	2.57±0.37	1.67±0.15^*∗*†^	0.036	2.52±0.12	2.43±0.13	0.088
(6) I certainly feel useless at times	2.45±0.18	1.91±0.23	0.071	2.43±0.14	2.28±0.17	0.055
(7) I feel that I'm a person of worth, at least on an equal plane with others	1.54±0.05	3.01±0.18^*∗*†^	0.024	1.53±0.10	2.11±0.07^†^	0.023
(8) I wish I could have more respect for myself	2.41±0.21	1.56±0.11^*∗*†^	0.015	2.39±0.13	2.17±0.03	0.096
(9) All in all, I am inclined to feel that I am a failure	2.73±0.20	1.31±0.12^*∗*†^	0.025	2.70±0.12	2.48±0.04	0.082
(10) I take a positive attitude toward myself	1.87±0.22	3.02±0.15^*∗*†^	0.019	1.83±0.11	2.09±0.11	0.091

Values represent mean ± SD.

Items 1, 3, 4, 7, and 10: A higher score indicates a better outcome; items 2, 5, 6, 8, and 9: A lower score indicates a better outcome.

^*∗*^Indicates a significant difference from the conventional group (*p*<0.05).

^†^Indicates a significant difference from the prescore (*p*<0.05).

## Data Availability

The data used to support the findings of this study are available from the corresponding author upon request.
